# Obtainment and confirmation of intergeneric hybrids between marguerite (*Argyranthemum frutescens* (L.) Sch.Bip.) and two *Rhodanthemum* species (*R. hosmariense* (Ball) B. H. Wilcox, K. Bremer & Humphries and *R. catananche* (Ball) B. H. Wilcox, K. Bremer & Humphries)

**DOI:** 10.5511/plantbiotechnology.23.0202a

**Published:** 2023-06-25

**Authors:** Hiroyuki Katsuoka, Naoya Hamabe, Chiemi Kato, Susumu Hisamatsu, Fujio Baba, Motohiro Taneishi, Toshiyuki Sasaki

**Affiliations:** 1 Izu Agricultural Research Center, Shizuoka Prefectural Research Institute of Agriculture and Forestry, 3012 Inatori, Higashiizu, Kamo, Shizuoka 413-0411, Japan

**Keywords:** *Argyranthemum*, gene marker, intergeneric hybridization, ovule culture, *Rhodanthemum*

## Abstract

*Argyranthemum frutescens* (L.) Sch.Bip. and *Rhodanthemum gayanum* (Coss. & Durieu) B. H. Wilcox, K. Bremer & Humphries are capable of hybridization. To expand flower color variation in this intergeneric hybrid group, we performed crosses using *A*. *frutescens* as the seed parent and *R*. *hosmariense* (Ball) B. H. Wilcox, K. Bremer & Humphries, *R. catananche* (Ball) B. H. Wilcox, K. Bremer & Humphries as the pollen parent. One plantlet was obtained from each cross between the white to pale pink-flowered *A*. *frutescens* and white-flowered *R*. *hosmariense*, and from a cross between the pink-flowered *A*. *frutescens* and cream to pale yellow-flowered *R. catananche*, via ovule culture. The cross with *R*. *hosmariense* produced an individual with white to pale pink ray florets, and the cross with *R*. *catananche* produced an individual with red ray florets. The flower and leaf shape of the progenies was intermediate between the parents, and other morphological traits were also characterized in the same manner. Morphological observations and a cleaved amplified polymorphic sequence marker-based determination, using the internal transcribed spacer region as a target for amplification and the restriction enzyme *Afl* II, revealed that both individuals are hybrids between *A*. *frutescens* and *R*. *hosmariense*, *R*. *catananche*. To the best of our knowledge, this is the first study to report that crossbreeding between *A*. *frutescens* (seed parent) and *R*. *hosmariense*, *R*. *catananche* (pollen parent) is possible. Moreover, further development of *Argyranthemum* breeding, especially that of a series of hybrid cultivars with different flower colors, is expected.

## Introduction

*Argyranthemum frutescens* (L.) Sch.Bip., commonly known as marguerite, is a perennial plant of the Asteraceae family and is widely cultivated for cut flowers and potted plants in Japan (Inaba 2019). The genus *Argyranthemum* is native to the Canary Islands and Madeira Islands, where there are 23 or 24 species with excellent characteristics, such as plant form and leaf shape (Bramwell and Bramwell 2001; Bremer and Humphries 1993; Inaba 2019; Press and Short 1994). In the commercial cultivation of flowering plants, it is essential to breed new cultivars with novel characteristics to stimulate demand and improve profitability. Owing to the relative ease of intra- and interspecific hybridization, *Argyranthemum* species have been actively bred and improved (Inaba 2019), and more than 200 cultivars have been registered or applied for variety registration in Japan. Alternatively, plants of the genus *Argyranthemum* have little genetic diversity in flower color and other characteristics, probably because their native habitat is limited to islands isolated from the continent (Inaba 2019; Ohtsuka and Inaba 2008). Although *A*. *frutescens* is categorized as a perennial plant, it sometimes fails to survive in the Japanese climate (high temperature in summer or frost damage in winter) (Inaba 2019). Hence, it is desirable to improve its adaptability to these conditions.

When breeding plants lacking diversity within the genus, introducing valuable traits by intergeneric hybridization is considered an effective breeding method (Knobloch 1972; Nakano and Mii 2008). During intergeneric hybridization in *A. frutescens*, embryo rescue facilitates attainment of progenies in crosses with *Chamaemelum nobile* (L.) All., *Glebionis coronaria* (L.) Cass. ex Spach, *Ismelia carinata* (Schousb.) Sch.Bip., and *Rhodanthemum gayanum* (Coss. & Durieu) B. H. Wilcox, K. Bremer & Humphries as pollen parents (Katsuoka et al. 2022; Muto et al. 2020a; Ohtsuka and Inaba 2008). Among these crosses, the hybrid cultivars “Bijoumum rosequartz (tentative name, former strain name “Izu No. 43”)” and “Bijoumum garnet (tentative name, former strain name “Izu No. 44”)” have been bred from intergeneric crosses between *A. frutescens* and *R*. *gayanum* (Katsuoka et al. 2021). They are now applying for variety registration in Japan. These two cultivars grow in a dwarf habitat and have vivid reddish-purple and red colored flowers (Katsuoka et al. 2021). Furthermore, farmers appreciate the superior heat and cold tolerances of these cultivars compared to existing *A. frutescens* cultivars (Katsuoka et al. 2021) and expect to expand their production in the future. Alternatively, selling various flower colors in one package has been standard in flower seedling sales and is considered an essential factor for favorable selling. Thus, we have performed crosses with this combination for more than six years to expand the variation of flower color of *A. frutescens*×*R. gayanum* hybrids; however, all obtained hybrids had pink or red flower color.

Meanwhile, there are 12 species of *Rhodanthemum* (Bremer and Humphries 1993), of which *R. hosmariense* (Ball) B. H. Wilcox, K. Bremer & Humphries, and *R. catananche* (Ball) B. H. Wilcox, K. Bremer & Humphries are cultivated in Japan, in addition to *R. gayanum*. As *R. hosmariense* and *R. catananche* have white and cream-to-yellow ray florets, respectively (Brickell 2016; Sutton 2001), we thought that crosses between these two species could be used to expand the variety of flower colors in hybrids of *A. frutescens* and *Rhodanthemum*. However, there have been no reports of hybridization of these combinations yet, and the flower color and other characteristics of the progeny are unknown. Here, to achieve profitable sales through the cultivation of a flower color series, we report on the obtainment of progenies using *A. frutescens* as the seed parent and *R. hosmariense*, *R. catananche* as the pollen parent by ovule culture and investigate the characteristics and hybridity of the obtained progenies.

## Materials and methods

### Plant materials

All plants used in the following experiments were cultivated as pot plants in the greenhouse under natural day length, no shading, and temperatures kept above 10°C.

### Intergeneric hybridization

Nine *A. frutescens* strains as seed parents (“08-23-1-X-1”, “08-23-1”, “P18-93-01”, “11-16-2”, “P12-26-1”, “P14-32-1”, “P16-62-03”, ‘Moonlight’, and “P19-34-02”) and three *Rhodanthemum* species (*R. hosmariense*, *R. catananche*, and *R. gayanum* [Control plants; intergeneric hybridization with *A. frutescens* via embryo culture has been reported (Muto et al. 2020a)]) as pollen parents were used in this study. All cross combinations are shown in Table 1. Crossings were performed as reported previously (Katsuoka et al. 2022) and were carried out on sunny mornings between March to July 2016 and 2021. About 60 disk florets of the second and third columns, from the exterior of the capitulum of *A. frutescens* were used for cross-pollination. Pollen from freshly opened *Rhodanthemum* flowers was transferred to the seed parent using a brush. After pollination, female flowers were enclosed in a paper bag.

**Table 1:** Table 1. Number of plants obtained from intergeneric crosses using *A. frutescens* as the seed parent and three *Rhodanthemum* species as the pollen parent via ovule culture.

Cross combination		No. of capitula pollinated	No. of ovule cultured	No. of germinating plants	No. of acclimatized plants	No. of plants that survived	No. of plants that bloomed
Seed parent (*A. frutescens*)	Pollen parent (*Rhodanthemum*)						
*A. frutescens*×*R. hosmariense*							
“08-23-1-X-1”	“20-Rh-1”	2	1	1	1	1	1
“08-23-1”	“20-Rh-1”	1	0	0	0	0	0
“P18-93-01”	“20-Rh-1”	3	0	0	0	0	0
*A. frutescens*×*R. catananche*							
“08-23-1”	‘Swan cream’	40	12	4	2	1	1
“11-16-2”	‘Swan cream’	1	0	0	0	0	0
“P12-26-1”	‘Swan cream’	2	0	0	0	0	0
“P14-32-1”	‘Swan cream’	2	0	0	0	0	0
“P16-62-03”	‘Swan cream’	22	1	0	0	0	0
‘Moonlight’	‘Swan cream’	5	0	0	0	0	0
“08-23-1-X-1”	‘Luna’	3	0	0	0	0	0
“P19-34-02”	‘Luna’	1	0	0	0	0	0
“08-23-1”	‘Retro cracker’	4	1	0	0	0	0
“P16-62-03”	‘Retro cracker’	2	0	0	0	0	0
*A. frutescens*×*R. gayanum*^z^							
“08-23-1-X-1”	‘African eyes’	36	7	4	3	3	1
“08-23-1”	‘African eyes’	10	2	1	1	1	0
“11-16-2”	‘African eyes’	17	0	0	0	0	0
“P16-62-03”	‘African eyes’	5	1	1	0	0	0
“P18-93-01”	‘African eyes’	3	0	0	0	0	0
‘Moonlight’	‘African eyes’	5	0	0	0	0	0
“08-23-1-X-1”	‘Elf pink’	16	21	11	9	6	5
“08-23-1”	‘Elf pink’	5	12	10	8	5	2
“11-16-2”	‘Elf pink’	5	0	0	0	0	0
“P12-26-1”	‘Elf pink’	10	1	1	1	1	1
“P16-62-03”	‘Elf pink’	5	7	4	2	1	1

^a^ Control: Combinations reported to be able to obtain intergeneric hybrids via embryo culture (Muto et al. 2020a).

### Ovule culture

A previously reported method (Katsuoka et al. 2022) was followed for ovule culture. Approximately three weeks after pollination, ovules were excised and cultured. Capitula were first dipped in 70% ethanol for a minute and then in 1% sodium hypochlorite solution for ten minutes for surface sterilization. After disinfection, ovules were rinsed twice with sterile distilled water for ovule rescue. Disc florets of the second and third columns used for cross-pollination were selected, and ovary walls were aseptically removed under a dissecting microscope. Mature ovules were immediately cultured in autoclaved culture tubes containing 10 ml of modified Murashige and Skoog (MS) medium (pH=5.8) (Murashige and Skoog 1962), consisting of half-strength mineral salts supplemented with 30 g·l^−1^ sucrose and 3 g·l^−1^ gellan gum. Cultures were maintained at 24°C under a 16 h light/8 h dark photoperiod. Ovule culture-derived plantlets showing normal growth were transplanted from culture tubes to plastic trays containing expanded vermiculite for acclimatization. Afterward, acclimatized plants were transplanted into polyethylene pots (10.5 cm in diameter) filled with commercial medium. Plants were subsequently grown under the same conditions as the parent plants and used for other experiments.

### Characterization of putative hybrids and their parents

The putative hybrids obtained from *A. frutescens*×*R. hosmariense* and *A. frutescens*×*R. catananche*, and their parents were used. Following a previously reported method (Ohtsuka and Inaba 2008), we examined the ray floret color, flower disk color, capitulum diameter, plant type (rounded, spreading, or upright), degree of branching, and leaf color of putative hybrids and their parents.

### DNA extraction and CAPS markers for discrimination

The putative hybrids obtained from *A. frutescens*×*R. hosmariense* and *A. frutescens*×*R. catananche*, and their parents were used. A DNeasy Plant Mini Kit (Qiagen, Hilden, Germany) was used to extract genomic DNA from 50 mg of young leaves collected from each plant. Hybridization was determined using genetic markers and performed according to a previously reported method (Muto et al. 2020a). The ITS region of nuclear ribosomal DNA was amplified using the primer pair 5′-AGAAATCGTAACAAGGTTTCCGTAGG-3′ (Zhao et al. 2010) and ITS4 5′-TCCTCCGCTTATTGATATGC-3′ (White et al. 1990). PCR amplification was performed using ExTaq DNA polymerase hot start version (Takara Co., Shiga, Japan). The PCR mixture (25 µl) contained 1 µl template genomic DNA, 2 µl of each primer (5 μM), 2.5 µl of 10×Ex Taq Buffer, 2 µl dNTP Mixture (each 2.5 mM), 0.125 µl Takara Ex Taq HS (5 U·µl^−1^), and 15.375 µl sterilized water.

PCR amplifications were performed in a SimpliAmp™ Thermal Cycler (Thermo Fisher Scientific Inc., Waltham, MA, USA) with initial denaturation at 95°C for 2 min, followed by 35 cycles at 94°C for 30 s, 56°C for 30 s, and 72°C for 1 min, with a final extension at 72°C for 5 min.

PCR products were subsequently digested with the restriction enzyme *Afl* II, which was used for hybrid determination (Muto et al. 2020a). The restriction enzyme reaction mixture (10 µl) contained 5 µl PCR amplification products, 0.5 µl restriction enzyme *Afl* II (10 U·µl^−1^), 1 µl 10×M buffer, 1 µl 0.1% BSA, and sterilized water to make 10 µl. The restriction enzyme reaction was performed at 37°C for 6 h. Mixtures containing 5 µl of enzyme-restricted reaction products and 1 µl bromophenol blue were resolved by electrophoresis (100 V for 40 min) on 1.5% (w·v^−1^) agarose gels in 1×TBE buffer and stained with ethidium bromide.

## Results

### Intergeneric hybridization

**Figure 1:**
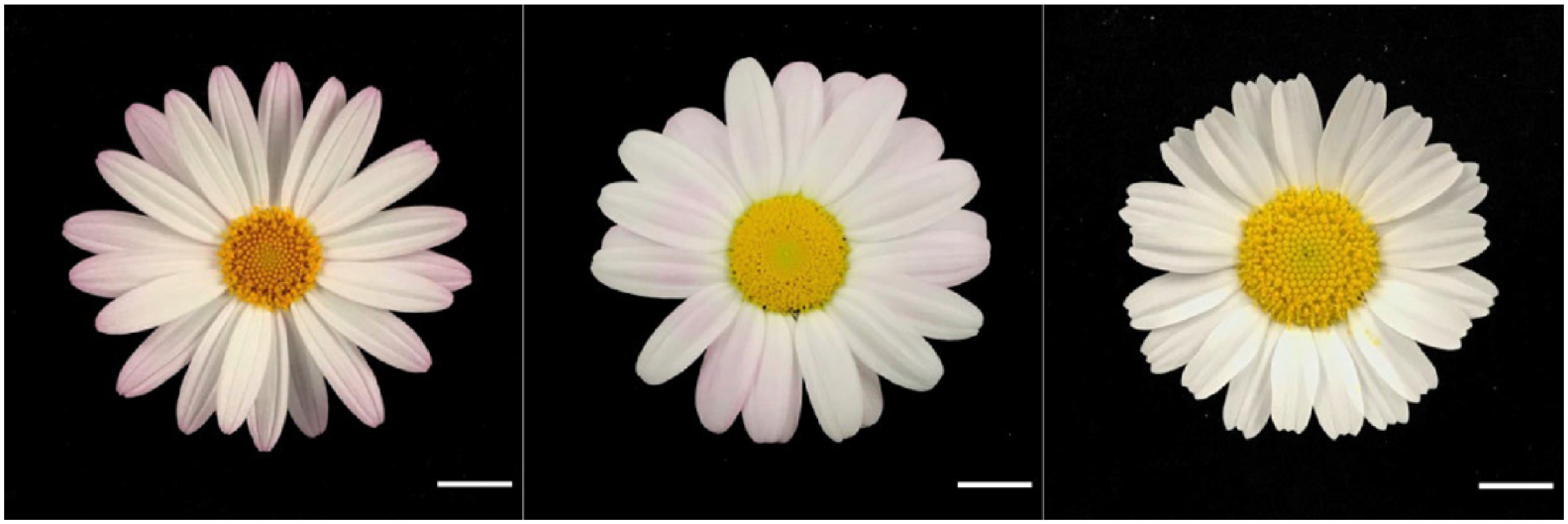
Figure 1. Phenotype of flowers (parents and the putative hybrid). Left: *A. frutescens* “08-23-1-X-1”, right: *R. hosmariense* “20-Rh-1”, middle: Putative hybrid “PH1” obtained from *A. frutescens* “08-23-1-X-1”×*R. hosmariense* “20-Rh-1”. Bar=1 cm.

**Figure 2:**
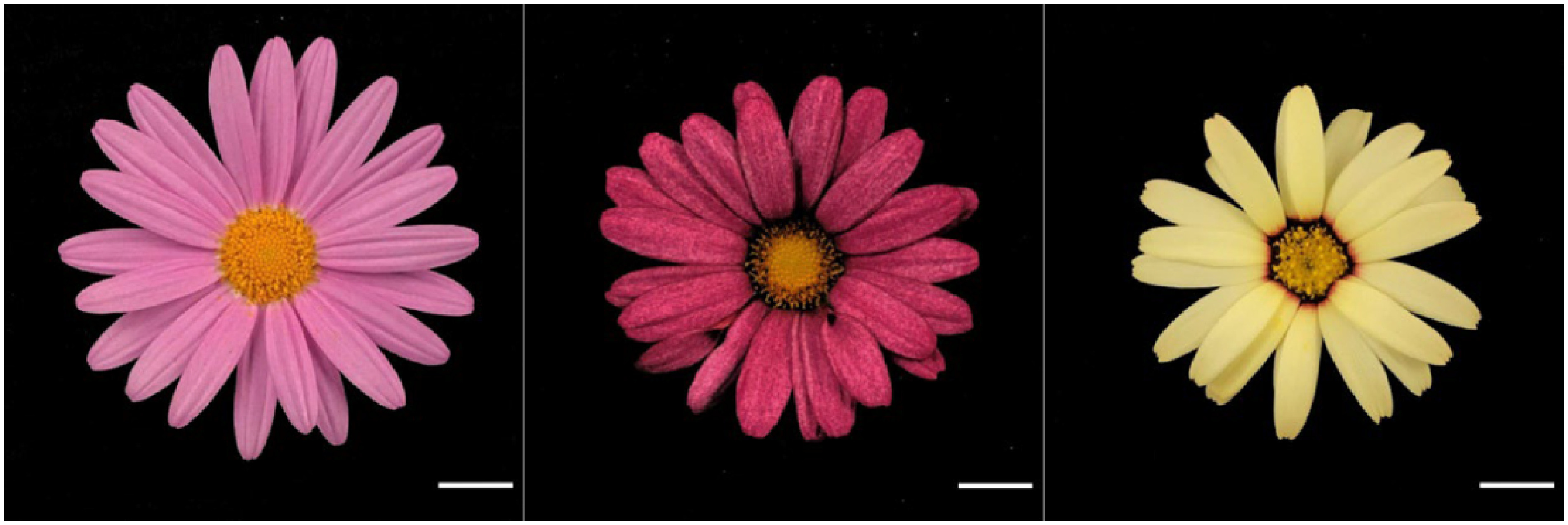
Figure 2. Phenotype of flowers (parents and the putative hybrid). Left: *A*. *frutescens* “08-23-1”, right: *R*. *catananche* ‘Swan cream’, middle: Putative hybrid “PH2” obtained from *A*. *frutescens* “08-23-1”×*R*. *catananche* ‘Swan cream’. Bar=1 cm.

**Figure 3:**
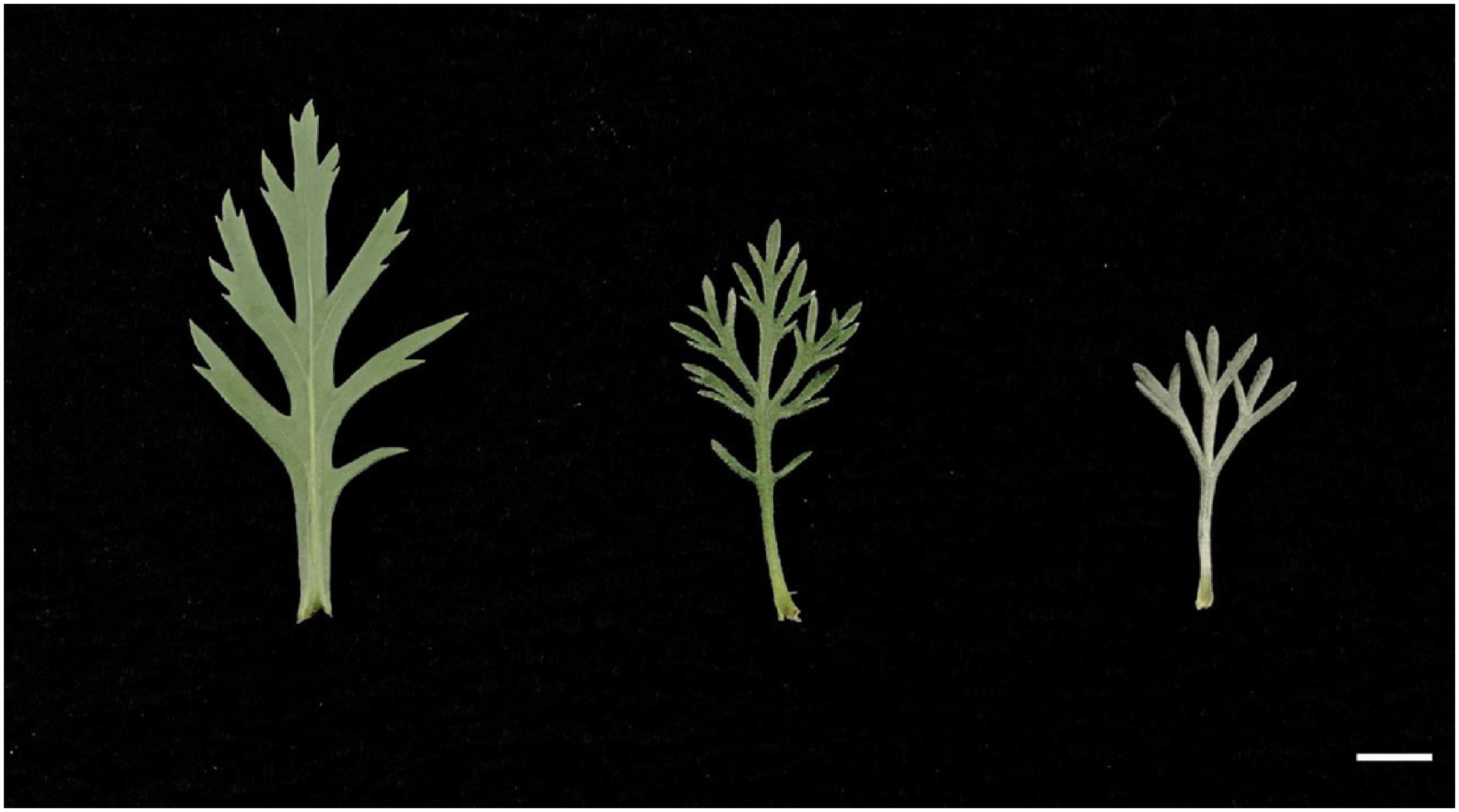
Figure 3. Phenotype of leaves (parents and the putative hybrid). Left:* A. frutescens* “08-23-1-X-1”, right: R. hosmariense “20-Rh-1”, middle: Putative hybrid “PH1” obtained from *A. frutescens* “08-23-1-X-1”×*R. hosmariense* “20-Rh-1”. Bar=1 cm.

**Figure 4:**
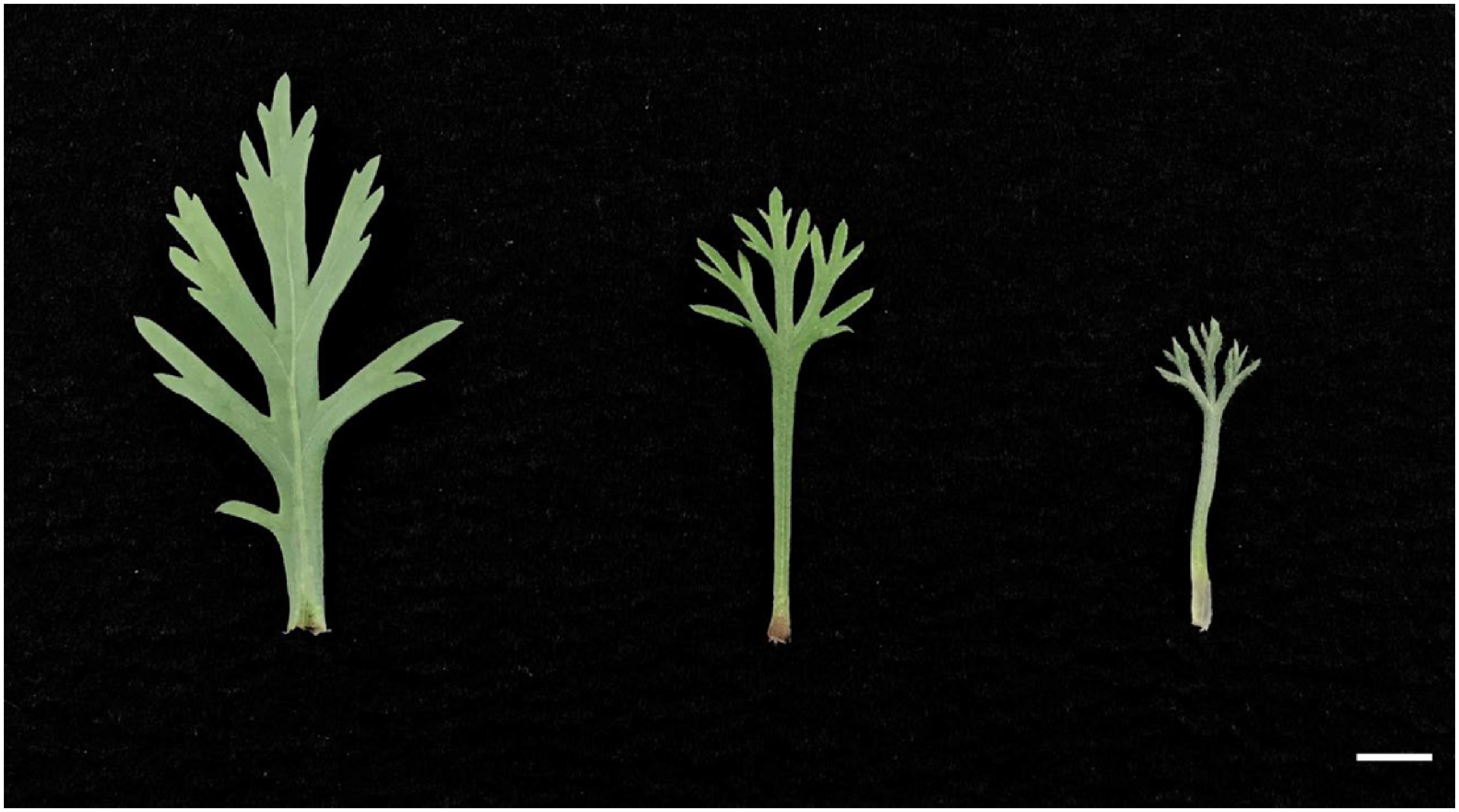
Figure 4. Phenotype of leaves (parents and the putative hybrid). Left: *A. frutescens* “08-23-1”, right: *R. catananche* ‘Swan cream’, middle: Putative hybrid “PH2” obtained from *A. frutescens* “08-23-1”×*R. catananche* ‘Swan cream’. Bar=1 cm.

Six capitula were used for the cross combination of *A. frutescens* and *R. hosmariense*, 82 for the cross combination of *A. frutescens* and *R. catananche*, and 117 for the cross combination of *A. frutescens* and *R. gayanum* (Table 1). The *Rhodanthemum* plants used as pollen parents produced many pollen grains and could attach sufficient amounts to the pistils of the disk florets. From crosses between *A. frutescens* and *R. hosmariense*, one well-developed ovule was obtained only from “08-23-1-X-1”×“20-Rh-1”, which bloomed after ovule culture and acclimatization (Table 1, Figure 1). From crosses between *A. frutescens* and *R. catananche*, “08-23-1”×‘Swan cream’ produced a relatively large number of well-developed ovules, but only one plantlet survived and flowered (Table 1, Figure 2). Based on morphological characteristics, such as plant posture, flower shape, and leaf shape intermediate between parents (Figures 1–4), the progeny from the crosses between *A. frutescens* and *R. hosmariense*, *R. catananche* were considered to be putative hybrids. On the other hand, the crosses between *A. frutescens* and *R. gayanum* yielded several plantlets from different combinations (Table 1). The morphological characteristics of these plants were similar to those previously reported (Muto et al. 2020a) and were intermediate between the parents (data not shown), so we treated them as hybrids between *A. frutescens* and *R. gayanum* (Table 1). The individual obtained from *A. frutescens* “08-23-1-X-1”×R. hosmariense “20-Rh-1” was named “PH1” and the individual obtained from *A. frutescens* “08-23-1”×*R. catananche* ‘Swan cream’ was named “PH2”.

### Characteristics of putative hybrids

The results are shown in Table 2. The putative hybrids, “PH1”, had white to pale pink ray florets, and “PH2” had red ray florets (Figures 1, 2). The color of the floral disk was yellow in “PH1” and yellow-orange in “PH2” (Figures 1, 2). The capitulum diameters of “PH1” and “PH2” were classified as “medium” at approximately 5 cm. This is intermediate in size between their parents. The plant type was round-shaped in all plants tested. More branching traits characterized both putative hybrids compared to their pollen parents. The leaf color of “PH1” and “PH2” was not the same as that of their parents, although both seed and pollen parents had gray-green leaf color (Figures 3, 4). The putative hybrids “PH1” and “PH2” were pollen-less individuals.

**Table 2:** Table 2. Morphological characterization of putative hybrids and their parents.

Plant name	Individual or strain name	Cross combination		Morphological characteristics^a^						Notes
		Seed parent (*A. frutescens*)	Pollen parent (*Rhodanthemum*)	Ray floret color^b^	Flower disk color	Capitulum diameter^c^ (cm)	Plant type	Branching^d^	Leaf color	
Putative hybrid	“PH1”	“08-23-1-X-1”	*R. hosmariense* “20-Rh-1”	White to pale pink	Yellow	Medium (5.4)	Rounded	Many	Dark green	Pollen-less
	“PH2”	“08-23-1”	*R. catananche* ‘Swan cream’	Red	Yellow orange	Medium (5.2)	Rounded	Many	Dark green	Pollen-less
*A. frutescens*	“08-23-1-X-1”			White to pale pink	Yellow orange	Medium (5.5)	Rounded	Moderate	Gray green	
	“08-23-1”			Pink	Yellow orange	Medium (5.7)	Rounded	Moderate	Gray green	
*R. hosmariense*	“20-Rh-1”			White	Yellow	Medium (5.2)	Rounded	Many	Gray green	Many pollen grains
*R. catananche*	‘Swan cream’			Pale yellow	Yellow	Medium (4.7)	Rounded	Many	Gray green	Many pollen grains

^a^Morphological characteristics are expressed according to the Marguerite Varietal Characteristic Systematic Investigation Standard specified by the Ministry of Agriculture, Forestry and Fisheries. ^b^Main color on the upper side of ray floret. ^c^Very small, below 3 cm; small, 3–4.5 cm; medium, 4.5–6 cm; large, >6 cm. Data in parentheses are averages of three capitula. ^d^Few, 0–2 branches per plant; moderate, 3–6 branches per plant; many, ≥7 branches per plant.

### Confirmation of hybridity using the CAPS method

**Figure 5:**
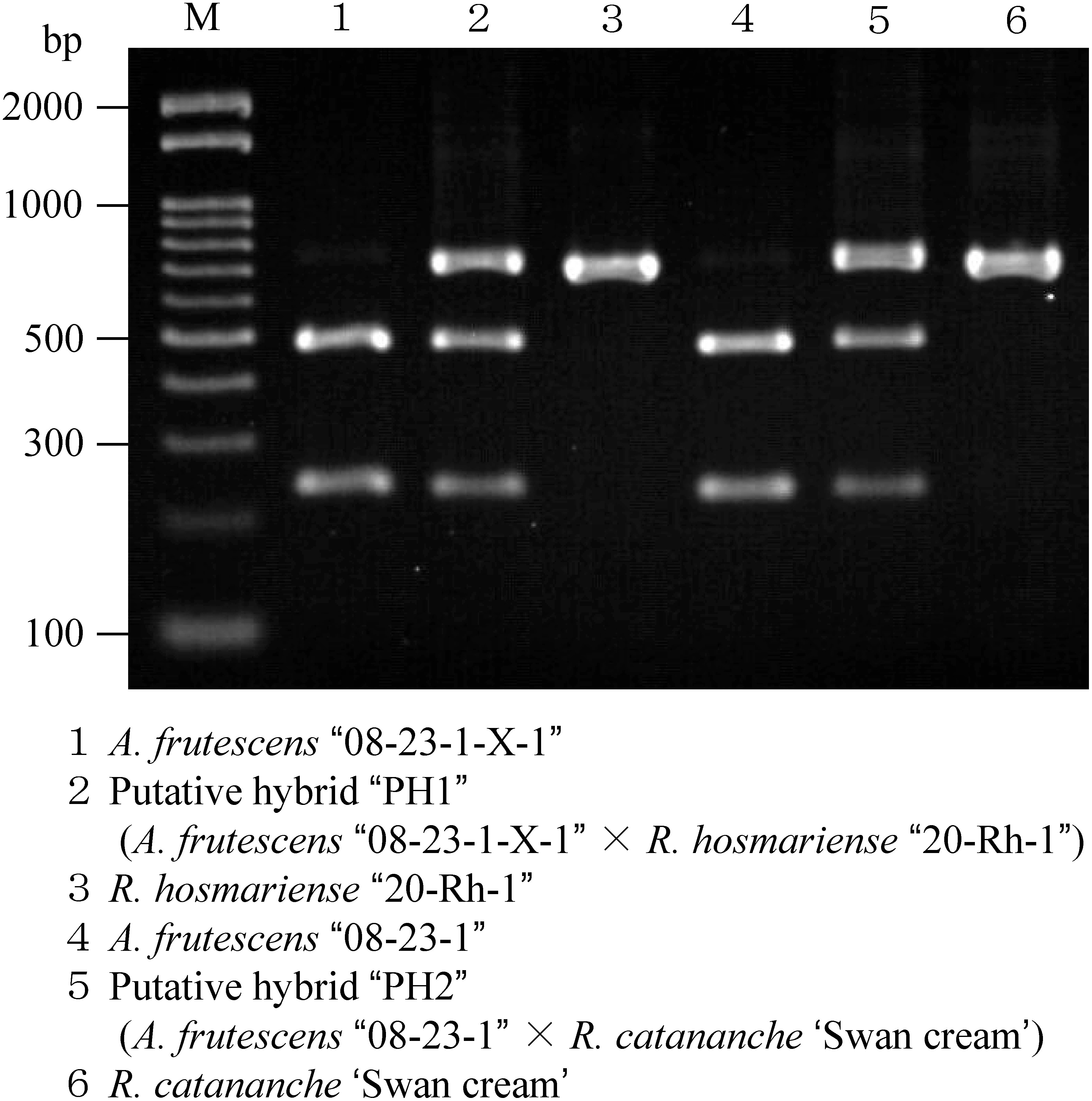
Figure 5. CAPS markers were used to confirm hybrids obtained from intergeneric hybridization between *A. frutescens* and *R. hosmariense* or *R. catananche*. Restriction enzyme *Afl* II was used. M indicates the marker lane.

Before restriction enzyme treatment, the PCR products formed a single band of approximately 740 bp for all samples. In the electrophoretic images of the restriction enzyme-treated PCR products, two specific bands were observed in *A. frutescens* and one in *Rhodanthemum* species at assumed positions. Furthermore, in putative hybrids obtained from each cross, three bands were detected in the same positions as those in the parents (Figure 5).

## Discussion

In this study, *A. frutescens* was used as the seed parent, and two *Rhodanthemum* species, *R. hosmariense* and *R. catananche*, with unknown cross compatibility, were used as pollen parents. The plantlets obtained by ovule culture were subjected to CAPS marker analyses and characterization to determine their hybridity. The CAPS method and morphological characteristics confirmed that the progenies were hybrids of both species (Table 2, Figures 1–5).

In intergeneric hybridization of *A. frutescens* as a seed parent, ovule culture enables the production of hybrids between *C. nobile*, *G. coronaria*, and *I. carinata* (Katsuoka et al. 2022; Ohtsuka and Inaba 2008), while embryo culture enables hybridization with *R. gayanum* (Muto et al. 2020a). In previous reports, crosses between *A. frutescens* and *R. hosmariense* were also performed; however, no hybrids were obtained (Muto et al. 2020a). In this study, we crossed several *A. frutescens* (seed parent) with one *R. hosmariense* strain, three *R. catananche* cultivars, or two *R. gayanum* cultivars (pollen parent). These *A. frutescens* strains are female fertile, as they have produced their progenies in previous intraspecific crosses. Although intraspecific crosses did not confirm the *Rhodanthemum* plants tested in this experiment, they all produced many pollen grains and are considered male fertile. After crossing these plants, ovules were cultured aseptically for obtaining hybrids. Although the number of capitulums crossed varied among cross combinations, hybrids were obtained from *A. frutescens* “08-23-1-X-1”×*R. hosmariense* “20-Rh-1” and *A. frutescens* “08-23-1”×*R. catananche* ‘Swan cream’, with one individual from each (Table 1). In several cross combinations, hybrids were also obtained from the cross between *A. frutescens* and *R. gayanum* (Table 1). Although the cross combinations that yielded these hybrids had a relatively high number of ovaries containing excisable ovules (Table 1), most of the approximately 60 disc florets per capitulum used in the cross-pollination were hollow and had no ovules in their ovaries. Previous reports suggest that the ease of obtaining hybrids in the intergeneric hybridization of *A. frutescens* varies depending on the plant species used as pollen parents, and hybrids between *R. gayanum* appear to be relatively more difficult to obtain than those between *C. nobile*, *G. coronaria*, and *I. carinata* (Katsuoka et al. 2022; Muto et al. 2020a; Ohtsuka and Inaba 2008). Although there are differences between embryo culture and ovule culture and in the cultivar and strain used in crosses, a comparison of the results of this study with existing reports suggests that hybrids are as likely or more difficult to be obtained when *R. hosmariense* and *R. catananche* are used as pollen parents as when *R. gayanum* is used as the parent (Katsuoka et al. 2022; Muto et al. 2020a; Ohtsuka and Inaba 2008). It is estimated that the more closely related a plant species is, the easier it will be to obtain posterity. Among the plant species that have been reported to hybridize with *A. frutescens*, *G. coronaria* and *I. carinata* are more closely related to *A. frutescens*, followed by *C. nobile*, and the genus *Rhodanthemum* is the most distantly related to *A. frutescens* (Francisco-Ortega et al. 1997; Oberprieler et al. 2007), which may have contributed to the difficulty in obtaining hybrids. Therefore, to produce many hybrids between *A. frutescens* and *Rhodanthemum* plants in the future, it is necessary to carry out hybridization and embryo rescue as efficiently as possible. Although the hybridization between *A. frutescens* and *R. gayanum* has been reported only using embryo culture, these results reveal that hybrids can also be produced using ovule culture (Table 1). Embryo culture in *A. frutescens* requires the removal of the thin, soft integument from the ovule after it has been extracted from the ovary. Because these organs are small and the process is complex, the fact that hybrids can be obtained using ovule culture may help in efficient breeding. Additionally, it would be useful to select parents from which hybrids can be easily obtained. In this research, we were able to obtain several hybrids between *A. frutescens* “08-23-1” or “08-23-1-X-1” and plants of the genus *Rhodanthemum* (Table 1). This “08-23-1-X-1” is a flower color mutant strain obtained from “08-23-1” irradiated with X-rays, so it is clear that “08-23-1” is a strain that has high cross-compatibility with the genus *Rhodanthemum*. Also, three *A. frutescens* strains used in this study, “P12-26-1”, “P16-62-03”, and “P18-93-01”, are the progeny from “08-23-1” or “08-23-1-X-1”. Among them, the crosses between “P12-26-1” or “P16-62-03” and *R. gayanum* produced one hybrid each, while none of the strains unrelated to “08-23-1” produced any hybrids or ovules (Table 1). This suggests that the cross-compatibility of “08-23-1” may be inherited. Unfortunately, three strains, “P12-26-1”, “P16-62-03”, and “P18-93-01”, did not have the same level of cross-compatibility as “08-23-1”, but it is expected that strains with higher cross-compatibility will be produced from the progeny of “08-23-1”. On the other hand, in pollen parent ‘Elf pink’, producing hybrids is easier than in ‘African eyes’, even in the same *R. gayanum* species (Table 1). In *R. hosmariense *and *R. catananche*, there are likely differences in cross-compatibility between strains, and therefore, it is necessary to search for suitable strains for hybridization. It may also be effective to cross *R. gayanum* ‘Elf pink’ with *R. hosmariense* or *R. catananche *to produce a parental line with high cross-compatibility. Additionally, for efficient production of hybrids, it would be necessary to investigate the reciprocal crossings, the period from the cross to ovule culture, the use of different media, and the possible effects of plant growth regulators.

This report’s two newly produced hybrids bloomed (Tables 1, 2, Figures 1, 2). The color of the ray floret of “PH1” was white to pale pink, similar to that of the seed parent (Figure 1). The color of the ray floret of “PH2” was red, different from that of both parents (Figure 2). The flower color of these hybrids is presumed to be due to the accumulation of anthocyanins, as in the previously reported *A. frutescens* and its intergeneric hybrids (Inaba 2019; Muto et al. 2020a). In hybrids between *A. frutescens* and *R. gayanum*, changes in the composition of anthocyanins in ray florets have been reported, with an increase in the ratio of pelargonidin content, which is rarely found in the petals of the parents (Muto et al. 2020a). Hybrids “PH1” and “PH2” may have the same altered anthocyanin composition. Also, the accumulation of carotenoids causes ray florets to turn yellow in *A. frutescens* (Inaba et al. 2006). The mechanisms that cause ray florets of *R. catananche *to appear pale yellow are unknown, but as with other Compositae, the accumulation of carotenoids is likely the main factor (Kishimoto et al. 2007). The expression of red flower color is the accumulation of anthocyanins and carotenoids, which may co-occur (Kishimoto et al. 2007). Therefore, the “PH2” of red flowers may contain carotenoids derived from *R. catananche*, and further analysis, including elucidation of the patterns of pigment inheritance, is expected. Other traits, except for leaf color, either in the seed parent, pollen parent, or both, were passed on to the hybrid (Table 2). In particular, the well-branched characteristic inherited from the *Rhodanthemum* species is considered useful for use as potted plants (Table 2). The hybrids “PH1” and “PH2” did not produce any pollen and were considered male-sterile (Table 2), while female fertility was not examined in this study. It is unclear whether the percentage of flowering individuals, heritability of morphological characteristics, including flower color, and male sterility are universally observed in *A. frutescens* and *R. hosmariense*, *R. catananche* hybrids, owing to the small number of individuals obtained in this study. Some of the hybrids between *A. frutescens* and *R. gayanum* obtained in this study did not flower (Table 1), and all hybrid individuals that did flower were male-sterile with no pollen production (data not shown). These characteristics observed in hybrids between *A. frutescens* and *R. gayanum* may be useful information for understanding the characteristics of hybrids obtained from crosses between *A. frutescens* and *R. hosmariense*, *R. catananche*, and for advancing the breeding of hybrids between these genera.

Methods for confirming hybrids include observation of morphological characteristics as well as chromosome observations, such as fluorescence in situ hybridization and genomic in situ hybridization (Chester et al. 2010; Younis et al. 2015), flow cytometry analysis (Dolezel 1997), and determination by DNA markers (Ballesfin et al. 2018; Kim et al. 2022; Olszewska et al. 2021; Premjet et al. 2019; Punjansing et al. 2021), are commonly used. In intergeneric hybrids of *Argyranthemum*, flow cytometry analysis revealed hybridity with *C. nobile* and *I. carinata* (Katsuoka et al. 2022; Ueda and Yamada 2005), and genetic markers have been reported to discriminate between hybrids of *C. nobile*, *G. coronaria*, *I. carinata*, and *R. gayanum* (Katsuoka et al. 2022; Morikawa et al. 2014; Muto et al. 2020a, 2020b; Ueda and Yamada 2005). In hybridization between *A. frutescens* and *R. gayanum*, flow cytometry analysis has not been performed to discriminate hybrids. Since flow cytometry is a simple and rapid method for determining hybridization, we preliminarily investigated the applicability of the previously reported method (Katsuoka et al. 2022), to determine hybrids between *A. frutescens* and *Rhodanthemum* plants. However, the peak positions of nuclear DNA content of *R. hosmariense*, *R. catananche*, and *R. gayanum*, which were used as pollen parents, all overlapped with those of *A. frutescens*, the pollen parent, and flow cytometry analysis could not determine their hybridity. The genome size of *A. frutescens* has been determined to be approximately 7 Gb (Suda et al. 2003), but the genome size of *Rhodanthemum* plants is unknown. Since the strain of *A. frutescens* used in this study was considered to be diploid based on its characteristics, and plants of the genus *Rhodanthemum* are diploid (Wagner et al. 2019), the genome size of the three species, *R. hosmariense*, *R. catananche*, and *R. gayanum*, was also estimated to be about 7 Gb. This knowledge will be useful in the future when it is necessary to confirm hybridization between *Rhodanthemum* plants and another genus or to obtain the whole genome sequence of *R. hosmariense*, *R. catananche*, and *R. gayanum* plants. Apart from flow cytometric analysis, the CAPS marker and sequence-characterized amplified region (SCAR) marker have been developed to discriminate hybrids between *A. frutescens* and *R. gayanum* (Muto et al. 2020a, 2020b). As a preliminary test, we applied the SCAR marker (Muto et al. 2020b) to determine hybridity for individuals obtained from *A. frutescens*×*R. hosmariense* and *A. frutescens*×*R. catananche*. However, this SCAR marker did not show good results, probably owing to differences in the polymerase, thermal cycler, and pollen parents used. The other method, the CAPS marker that uses the ITS region as a target for amplification and the restriction enzyme *Afl* II (Muto et al. 2020a), was used in this study. Based on comparison of already known sequences of the ITS region in the genus *Rhodanthemum* (accession No. MK481460, MK481483, MK481530 (Wagner et al. 2019); MN182353, MN182364, MN182386 (Wagner et al. 2020)), it was assumed that it could be used for the detection of hybrids even when the pollen parents are *R. hosmariense* and *R. catananche*. Analysis of electrophoretic images confirmed that amplified products treated with restriction enzymes formed specific bands derived from the genetic background (Figure 5). It was also possible to discriminate hybrids using differences in the number of these bands (Figure 5). Although the number of samples tested was small, the same pattern of electrophoretic images was found in two *Rhodanthemum* species (Figure 5), and the same restriction enzyme, *Afl* II, can be used to determine intergeneric hybridization between *A. frutescens* and *C. nobile* (Katsuoka et al. 2022). Hence, this method is expected to be a comprehensive marker for determining hybridization between *A. frutescens* and *Rhodanthemum* plants, enabling early selection at the early seedling stage.

This is the first study to report that *A. frutescens *(seed parent) and *R. hosmariense*, *R. catananche* can be crossed. Although generating hybrids with pure white or yellow ray florets was the main purpose of breeding, these were not obtained in this study. It is thought that this result will contribute to the expansion of flower color variation in hybrids between *A. frutescens* and *Rhodanthemum*. However, only pink or red flowers have been obtained so far (Katsuoka et al. 2021; Muto et al. 2020a). “PH1” was whiter in flower color than the hybrids between *A. frutescens* and *R. gayanum* obtained in previous reports and in this study (Figure 1) (Katsuoka et al. 2021; Muto et al. 2020a). For breeding hybrids with pure white flower color, using *R. hosmariense*, which has white flowers as the norm (Brickell 2016; Sutton 2001), is probably more suitable than *R. gayanum*, which is a pink-flowered species (Brickell 2016; Sutton 2001). For breeding yellow-flowered hybrids, using *R. catananche* with cream to pale yellow ray florets is an effective method, although it would be better to analyze the pigment components described above. In this test, “P18-93-01”, which has pure white ray florets, and “P16-62-03”, which has yellow ray florets, were used as seed parents; however, they did not produce any progeny in the crosses between *R. hosmariense* or *R. catananche*, respectively. Future breeding of hybrids with combinations between pure white-flowered *A. frutescens* and *R. hosmariense*, and yellow-flowered *A. frutescens* and *R. catananche*, is expected. Alternatively, the color of the flower disc and the ray floret is an important factor in flower appreciation. While flower discs of the species *R. gayanum* and its hybrids “Bijoumum rosequartz” and “Bijoumum garnet” were brown (Brickell 2016; Katsuoka et al. 2021; Sutton 2001), the hybrids obtained in this study had yellow or yellow-orange flower discs (Figures 1, 2). Using *R. hosmariense* and *R. catananche*, which generally have yellow flower discs (Brickell 2016), as pollen parents has expanded the color variation of hybrid disk florets (Figures 1, 2). Regarding traits other than flower color, the fact that *R. hosmariense* and *R. catananche* were available for crosses may allow for introducing traits superior to *R. gayanum* into *A. frutescens*. In interspecific and intergeneric crosses, hybrids show intermediate morphology between the parents (Nakano and Mii 2008). Indeed, the capitula of the hybrids in this study were intermediate in size to those of the parents (Table 2). As the capitulum diameters of *R. hosmariense* and *R. catananche* are typically larger than that of *R. gayanum* (Brickell 2016; Sutton 2001), the use of both species as hybrid parents should lead to larger capitulum diameters in hybrids than when using *R. gayanum* as pollen parents. *R. hosmariense *is easier to grow and has a longer flowering period than *R. gayanum* (Sutton 2001). The hybrids “Bijoumum rosequartz” and “Bijoumum garnet”, obtained from *A. frutescens*×*R. gayanum*, begin flowering around late January or early February (Katsuoka et al. 2021). Hybridization with *R. hosmariense* is expected to produce hybrids that are easier to grow, flower earlier, and have a longer flowering period than existing hybrids. In addition, *Rhodanthemum* plants are considered to be more cold-tolerant than *A. frutescens*. In commercial growth, the minimum temperature for marguerite cultivation is reported to be 10°C (Inaba 2019), while the minimum temperature for the cultivation of *Rhodanthemum* is 5°C (Saeda et al. 2021), and hybrids may also be able to grow at lower temperatures compared to *A. frutescens*. In this study, we did not examine flowering time, test for cold tolerance, or measure plant height or flower diameter, which are important for potted plants. In the future, we will elucidate the growth and flowering characteristics of hybrid plants obtained, and based on this research, we will promote crosses between *A. frutescens* and the *Rhodanthemum* species and early selection using CAPS markers, to breed a series of hybrid cultivars in different flower colors.

## Abbreviations

CAPS: cleaved amplified polymorphic sequence
ITS: internal transcribed spacer
PCR: polymerase chain reaction

## Note

A part of this article was presented at the 2022 Autumn Meeting of the Japanese Society for Horticultural Science.
